# A Floating‐Gate Photoelectric Synaptic Transistor Utilizing BP/PO_x_/WSe_2_ Heterostructure for Neuromorphic Visual Processing

**DOI:** 10.1002/advs.202510063

**Published:** 2025-09-14

**Authors:** Yuxuan Zeng, Wenxing Lv, Zehai Hou, Weihua Huang, Zhipeng Yu, Zishuo Han, Rongbin Zhan, Tianle Zeng, Yawen Luo, Yu Lin, Weiming Lv, Bin Fang, Zhongming Zeng, Lianbo Guo

**Affiliations:** ^1^ School of Optical and Electronic Information Huazhong University of Science and Technology Wuhan 430074 China; ^2^ School of Software Engineering Huazhong University of Science and Technology Wuhan 430074 China; ^3^ Nanofabrication facility Suzhou Institute of Nano‐Tech and Nano‐Bionics Chinese Academy of Sciences Suzhou 215123 China; ^4^ Physics Laboratory, Industrial Training Center Shenzhen Polytechnic University Shenzhen Guangdong 518055 China

**Keywords:** 2D vdW materials, floating‐gate transistor, neuromorphic computing, photoelectronic synaptic device

## Abstract

Artificial intelligence (AI) is constrained by the high energy consumption of von Neumann architectures and the limited scalability of traditional silicon‐based synapses. Two‐dimensional (2D) van der Waals (vdW) materials, with their atomic‐scale thickness, tunable electronic properties, and ease of heterogeneous integration, offer a promising platform for next‐generation neuromorphic hardware. Here, the authors report a vdW floating‐gate transistor (BP/PO_x_/WSe_2_) with a high on‐off current ratio (≈10^5^) and a large memory window (73 V), benefitting from the optimized interface band alignment via 2D heterostructure engineering. Key synaptic functionalities are demonstrated, including short‐term plasticity (STP), long‐term plasticity (LTP), and electro‐optical dependent plasticity, short‐term paired‐pulse facilitation (PPF), and long‐term potentiation/depression (LTP/D). Notably, the device mimics human visual memory under optical stimuli while achieving ultralow energy consumption (10 pJ per synaptic event), outperforming most reported photoelectronic synaptic devices. Furthermore, a two‐path convolutional neural network (CNN) is introduced that synergistically merges optical and electronic inputs, which enables efficient feature extraction and weight updating, and achieves 96.9% accuracy in the Labeled Faces in the Wild (LFW) face recognition task. The work presents a promising approach for neuromorphic electronics, paving the way for energy‐efficient vision processing in edge AI applications.

## Graphical abstract

In this paper, a novel floating gate transistor (BP/POx/WSe_2_) is developed, which enables rich synaptic functionality under optoelectronic conditions and can mimic human visual memory. By introducing a two‐path convolutional neural network that synergistically fuses optical and electronic inputs, it can achieve efficient feature extraction and weight updating, and achieve 96.9% accuracy in the (LFW) face recognition task.

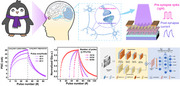

## 1 Introduction

With the rapid development of AI technology, the conventional von Neumann architectures face a serious challenge in energy efficiency when processing massive amounts of data because of the physical separation of memory from computation.^[^ ^1^, ^2^, ^3^ ^]^ A striking example reveals that ChatGPT consumes ≈564 MWh of electricity to process 195 million daily requests, substantially constraining the broad implementation of AI technologies.^[^ ^4^ ^]^ Brain‐inspired neuromorphic computing, with advantages in terms of high energy efficiency, high fault tolerance, and low operational latency, brings a promising approach to this challenge.^[^ ^5^, ^6^, ^7^, ^8^, ^9^, ^10^ ^]^ In the neuromorphic system, artificial synaptic devices are considered the key units, and their properties are essential to achieve efficient, high‐precision computation. In general, the existing device structures, such as two‐terminal memristor and three‐terminal transistor, are designed to realize the synaptic functions.^[^ ^11^ ^]^ Among them, three‐terminal transistor has attracted considerable attention owing to their nondestructive weight‐update behavior and diverse modulation of synaptic behavior, benefiting from the completely separated terminals for reading (drain) and writing (gate).^[^ ^7^, ^8^, ^9^, ^10^, ^12^, ^13^, ^14^, ^15^ ^]^ In particular, three terminal floating‐gate transistors with charge‐storage functions have been extensively investigated in the field of electronic or optoelectronic artificial synapses based on the vdW heterojunction of 2D materials due to their unique structural and physical properties.^[^ ^16^, ^17^ ^]^ For instance, Liao's group developed a ReS_2_/hBN/graphene heterostructure floating‐gate device, which successfully realized a neuromorphic vision system capable of performing multi‐target recognition tasks under a variety of stimuli, demonstrating efficient use of limited neural computing resources.^[^ ^14^ ^]^ Zhang's group achieved 97.7% accuracy in MNIST pattern recognition through optimized synaptic weight modulation in their MoS_2_/hBN/monolayer graphene architecture, which indicates the exceptional potential of 2D heterostructures in artificial synaptic applications.^[^ ^18^ ^]^

WSe_2_ has established itself as an exceptional channel material for synaptic transistors, distinguished by its broadband spectral responsivity, superior charge carrier mobility, and robust electrical stability across diverse operational conditions.^[^ ^19^, ^20^, ^21^, ^22^ ^]^ In contrast, black phosphorus (BP) has garnered significant attention for its extraordinary hole‐dominated transport properties, yet remains plagued by spontaneous ambient oxidation that critically undermines its practical viability.^[^ ^23^, ^24^, ^25^ ^]^ Paradoxically, however, this intrinsic chemical vulnerability has been strategically exploited for functional innovation. Notably, Walia et al. have successfully demonstrated light‐activated logic operations and neuromorphic computing capabilities by leveraging BP's oxidation‐sensitive characteristics.^[^ ^26^, ^27^, ^28^ ^]^ Nevertheless, extended atmospheric exposure inevitably induces progressive structural degradation and irreversible performance deterioration in BP‐based devices.^[^ ^24^ ^]^ Through strategic device engineering and proper encapsulation strategies, this stability challenge can be effectively addressed while preserving functional versatility. Liao et al. designed a MoS_2_/BP/MoS_2_ multilayer heterostructure, which significantly enhanced the light‐detection sensitivity and nonvolatile storage performance of the device by introducing a BP/PO_x_ interfacial layer. Based on density‐functional theory calculations and experimental verification, they clarified that PO_x_ works as an optoelectronic “valve” for electron injection at the interface.^[^ ^29^, ^30^ ^]^ During the fabrication process of floating‐gate devices, compared with traditional dielectric materials (such as A_2_O_3_), the spontaneous heterostructure of BP/PO_x_ not only simplifies the device fabrication steps but also avoids the secondary damage of BP. These advances highlight the great potential of integrating BP/PO_x_ heterogeneous structures into floating‐gate architectures and using them for brain‐inspired computational applications.

In this study, we propose an innovative floating‐gate synaptic transistor with a BP/PO_x_/WSe_2_ heterostructure in which spontaneously oxidized BP‐derived phosphorus oxides (PO_x_) are strategically integrated into a hybrid floating‐gate/dielectric layer stack. The device exhibits a high on‐off current ratio approaching 10^5^ and an ultra‐large memory window of 73 V. The synaptic features such as STP, LTP, PPF, and bi‐directional weight updating (LTP/D) are effectively accomplished by using a series of electric or photoelectric stimulation. Importantly, using the illumination of 365 nm wavelength light, this synaptic transistor can well recognize the mimicry of human visual memory and ultra‐low energy consumption (≈10 pJ) by reducing the read (drain‐source) voltage (*V*_ds_). Moreover, we have innovatively developed a dual‐path CNN algorithm that can synergistically process optical pulse sequences and electrical bias parameters, which effectively improves the feature processing efficiency and thus achieves up to 96.9% accuracy in the LFW face recognition task. Our work not only establishes a material‐structure co‐design paradigm for photoelectronic synaptic devices but also provides practical hardware solutions for the development of visually enhanced neuromorphic computing systems, especially in bio‐inspired AI applications.

## 2 Results and Discussion

### 2.1 Material and Device

**Figure 1:**
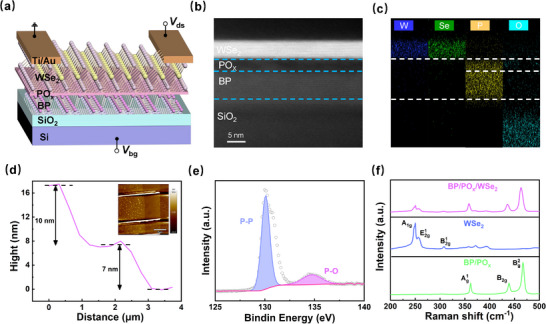
a) Schematic of the WSe_2_/PO_x_/BP floating‐gate transistor structure. b) HRTEM diagram of the device structure. c) EDS element mapping of the device, with a scale of 5 nm. d) Thickness diagram of BP/PO_x_ and WSe_2_ flakes measured by AFM. e) The XPS spectrum of BP exposed in the air for 20 min. f) Raman spectrograms of single WSe_2_, BP/PO_x,_ and WSe_2_/PO_x_/BP heterojunction regions.

The schematic diagram of the fabricated floating‐gate transistor is illustrated in **Figure** **1**a, where WSe_2_, PO_x_, BP, SiO_2_, and Si serve as the channel, the tunneling barrier, the floating‐gate, the control gate dielectric, and the control gate, respectively. The fabrication process flow is explained in detail in the experimental section. An important innovation in the device structure is the utilization of BP/PO_x_ heterogeneous structures as the floating‐gate architectures, in which the charge transfer to and from the PO_X_ layer results in the synaptic behaviors of the device. The cross‐section of the device and the composition of the PO_x_ layer are characterized by high‐resolution transmission electron microscopy (HRTEM). The HRTEM image in Figure 1b clearly shows that there is ≈3 nm amorphous layer between the BP and WSe_2_. The energy dispersive x‐ray spectroscopy (EDS) elemental mapping of the corresponding cross‐sectional regions in the HRTEM is shown in Figure 1c, which confirms that the interfacial amorphous layers are phosphorus oxides as both P and O elements are present between the BP and WSe_2_. Figure S1 (Supporting Information) shows an optical image of the device, where the cyan, yellow, and red regions correspond to BP, WSe_2_ and the electrode pins used in the experiment, respectively. The thickness of the device measured by atomic force microscopy (AFM) is shown in Figure 1d, where the thicknesses of BP/PO_x_ and WSe_2_ are 10 and 7 nm, respectively. X‐ray photoelectron spectroscopy (XPS) of BP exposed to air for 20 min, as shown in Figure 1e, shows that the P 2p nuclear level binding energy displays two distinct features corresponding to P─P at 130.1 eV and P─O at 134.8 eV, which is in agreement with the results in the literature,^[^ ^27^, ^31^, ^32^ ^]^ which is further evidence for the PO_x_ exists. The Raman spectra are shown in Figure 1f, and the characteristic peaks are in agreement with the previously reported results.^[^ ^33^, ^34^, ^35^ ^]^

### 2.2 Characterization of Basic Electrical Properties

**Figure 2:**
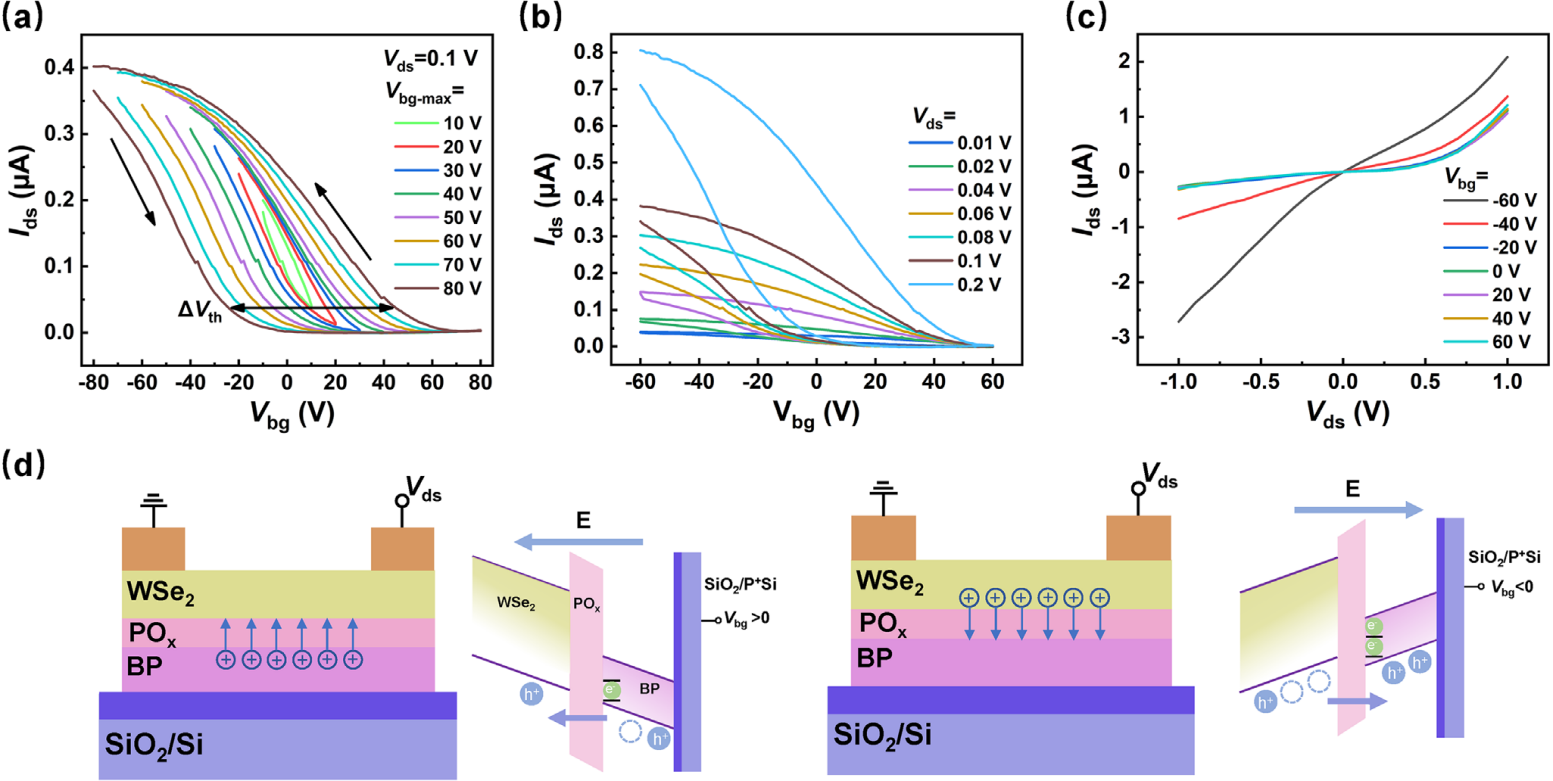
a) The *I*_ds_‐*V*_bg_ curve of the device, with *V*_ds_ fixed at 0.1 V and *V*_bg‐max_ varying from 10 to 80 V. The black arrow indicated the direction of the hysteresis loop. b) The *I_ds_*‐*V_bg_* curves with different *V*_ds_ values. c) Output curve of the device, *V*_ds_ sweeps from ‐1 to 1 V and *V*_bg_ varies from ‐60 to 60 at 20 V intervals. d) Energy band diagrams of tunneling mechanisms at different values of *V*_bg._

The transfer characteristics of the BP/PO_x_/WSe_2_ device is displayed in **Figure** **2**a. When sweeping the control back‐gate voltage (*V*_bg_) applied to the Si back gate, the drain‐source current (*I*_ds_) through the WSe_2_ channel was monitored. As indicated by the black arrows, a wider hysteresis window (referred to as a “memory window”) can be obtained for a larger sweeping range. Figure S2 (Supporting Information) is the logarithmic form graph of Figure 2a, which shows that the on/off current ratio of this device is ≈10^5^. Additionally, the memory window (Δ*V*_th_), defined as the difference in threshold voltage, is displayed as a function of the maximum *V*_bg_ sweeping range (*V*_bg‐max_) in Figure S3 (Supporting Information), and it shows that Δ*V*_th_ increases with *V*_bg‐max_, indicating that the charge‐trapping process in the WSe_2_/PO_x_/BP device can be effectively modulated by controlling the *V*_bg_. When the sweeping voltage *V*_bg_ = ± 80 V, the memory window of the device has reached 73 V. However, the memory window shows no appreciable variation when applying different drain‐source voltage *V*_ds_, as shown in Figure 2b, which revealed that the charge‐trapping process can be obviously modulated in the vertical direction of the heterostructure rather than the planar direction of the channel layer. This is also illustrated in Figure S4 (Supporting Information) by the comparative results of the *I*_ds_‐*V*_bg_ curves of the device for the same *V*_bg‐max_ value but different *V*_ds_ conditions. The reliability of the *V*_bg_‐controlled electric memory was investigated based on retention and cyclic writing/erasing endurance tests. Figure S5 (Supporting Information) shows that the device has reliable retention characteristics exceeding 11 000 s, demonstrating its excellent nonvolatile storage performance. In Figure S6 (Supporting Information), the device's endurance capability was obtained by running 1000 cycles on the device's transfer characteristic curve under the conditions of *V*_ds_ = 0.1 V and *V*_bg‐max_ = 50 V. The highest on (*V*_bg_ = ‐50 V) and off (*V*_bg_ = 50 V) states maintained a large on/off current ratio of ∼10^3^, and the current decay in all states was less than 10%, indicating the device's robustness.

To gain a deep insight into the storage mechanism of the BP/PO_x_/WSe_2_ device, we measured the electrical transport properties of both BP/PO_x_/WSe_2_ and individual WSe_2_ field effect transistors. The output and transfer characteristics of individual WSe_2_ field effect transistor (FET) were displayed in Figures S7 and S8 (Supporting Information), respectively. It is evident that the individual WSe_2_ FET exhibits a Schottky contact between WSe2 and electrodes (Figure S7, Supporting Information), simultaneously, shows a p‐type conductive behavior (Figure S8, Supporting Information). Interestingly, the output characteristics of BP/PO_x_/WSe_2_ device shown in Figure 2c demonstrate obvious diode functionality. Then, we considered the monitored current in the positive *V*_ds_ region to further investigate the specific transport mechanism. Figure S9 (Supporting Information) illustrates the value (ln(*I*_ds_ */V*_ds_^2^)) as a function of *V*_ds_^−1^ in the positive *V*_ds_ region, which demonstrates a Fowler‐Nordheim (FN) tunneling process in the large *V*_ds_ (> 0.5 V) regionand a direct tunneling in the small *V*_ds_ (< 0.5 V) region.^[^ ^36^ ^]^ However, as the *V*_bg_ increases to ‐60 V, charge transport is dominated by direct tunneling. Considering that the output characteristics of individual WSe_2_ channel show no significant variation with *V*_bg_, it is reasonable to suspect that tunneling occurs at the vertical interface of heterostructure (similar to the case of MoS_2_/BP/MoS_2_^[^ ^29^ ^]^), rather than within the WSe_2_ channel itself. Therefore, the storage mechanism of the BP/PO_x_/WSe_2_ device can be understood as depicted in Figure 2d. When a negative *V*_bg_ is applied to the back gate, a number of holes in WSe_2_ channel are induced and readily tunnel directly from WSe_2_ through the PO_x_ layer into BP due to the small read voltage (*V*_ds_ = 0.1 V). Subsequently, these holes are stored in the BP floating‐gate, confined by the interfacial tunneling barrier formed by the PO_x_ layer. The stored holes in BP is equivalent to a partial positive gate voltage, effectively depleting the WSe_2_ channel. This results in low conductance in the WSe_2_ channel at *V*_bg_ = 0 V and a negative shift of *V*th. Conversely, when a positive *V*_bg_ is applied to the back gate, holes can be pushed back to the WSe_2_ layer from the BP floating‐gate with the help of the electric field generated by the positive *V*_bg_, resulting in a high conductance in the WSe_2_ channel and positive shift of *V*_th_.

### 2.3 Electrically Modulated Synaptic Properties

**Figure 3:**
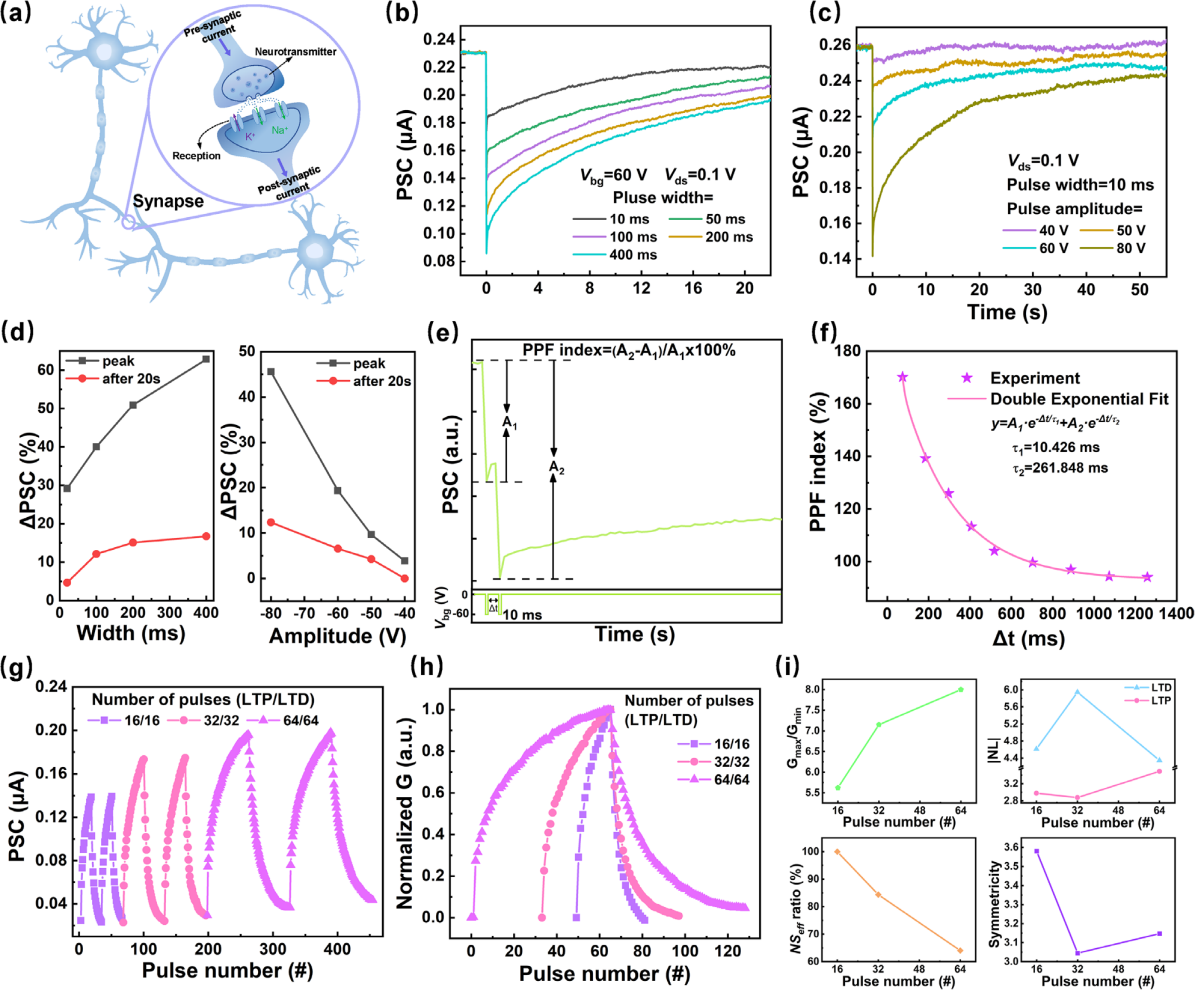
a) Schematic diagram of biological synaptic messaging. b) Single‐pulse electrical stimulation of PSC at fixed *V*_ds_ of 0.1 V and *V*_bg_ pulse amplitude of 60 V with different *V*_bg_ pulse widths from 10 to 400 ms. c) Single‐pulse electrical stimulation of the PSC at a fixed *V*_ds_ of 0.1 V and varying *V*_bg_ amplitudes of 40 V to 80 V while maintaining a *V*_bg_ pulse width of 10 ms. d) Effect of varying pulse width and amplitude on synaptic weight. e) PSC amplitudes induced by a pair of gate pulses (A_1_ and A_2_). f) PPF index plotted as a function of Δt, fitted by a biexponential decay model (pink line). g) Two‐cycle LTP/D characteristic curves for different pulse numbers. h) Normalized conductance of LTP/D at different *V*_bg_ pulse numbers. i) G_max_/G_min_, |NL|, *NS*_eff_, and symmetricity values at different pulse numbers.

Based on the electrical properties established above, the synaptic behavior of this device under electrical modulation was further investigated. In the biological nervous system, a synapse is the connection between neurons that enables their electrical or chemical signals transmission. The connectivity between neurons, named synaptic weight, can be strengthened or weakened by the number of released neurotransmitters as shown in **Figure** **3**a.^[^ ^36^, ^37^, ^38^, ^39^ ^]^ Correspondingly, in our three‐terminal BP/PO_x_/WSe_2_ floating‐gate device, the electrical pulse from the Si back gate or the optical illumination is considered as the presynaptic voltage spike, which leads a change of synaptic weight. The drain‐source current *I*_ds_ in WSe_2_ channel plays the role of postsynaptic current (PSC). By adjusting the parameters of the pulses (e.g., width, amplitude, frequency, etc.), precise control of synaptic weights can be realized, thus simulating the learning and memory functions of biological synapses.^[^ ^40^, ^41^ ^]^

In the biological nervous system, changes in synaptic weights between presynaptic and postsynaptic neurons were referred to as synaptic plasticity, which includes STP and LTP.^[^ ^42^ ^]^ STP usually lasts from a few milliseconds to a few seconds, and it was a temporary modulation mechanism for neural information processing, while LTP usually lasts from a few minutes to a few hours.^[^ ^43^ ^]^ As can be seen in Figure 3b, when a fixed *V*_ds_ = 0.1 V and *V*_bg_ = 60 V were applied, the range of variation of the PSC increased with the duration of the applied pulse. As shown in Figure 3c, under the condition of *V*_ds_ = 0.1 V and a pulse width of 10 ms, when a small amplitude (40 V) pulse was applied, the PSC peak was small and returned to the initial state within a short period of time, exhibiting STP characteristics. When a large amplitude (80 V) pulse was applied, the PSC peak was high and held for a long time, exhibiting LTP characteristics. Even long after the end of the pulse, the synaptic weights still failed to recover to the initial state, a phenomenon that could be modeled as LTP, where the decay time corresponds to the synaptic weights. Figure 3d clearly shows that the enhancement of synaptic weights updating can be controlled by adjusting the amplitude and width of the *V*_bg_ pulse. Thus, the transition of the device from STP to LTP can be achieved by adjusting the amplitude of *V*_bg_.

PPF is a manifestation of STP and refers to the relevant changes in synaptic responses after the first and second consecutive stimuli.^[^ ^44^ ^]^ In general, the PPF index is defined as = (A_1_‐A_2_)/A_1_×100%, where A_1_ and A_2_ are the values of the first and second PSCs, respectively.^[^ ^45^ ^]^ As shown in Figure 3e, the PPF behavior is generated by applying a pair of positive gate pulses (60 V, duration 10 ms). As shown in Figure 3f, the extracted PPF index as a function of the time between the two pulses was fitted using a biexponential decay function to calculate the obtained relaxation time constants (τ_1_ = 10.426 ms, τ_2_ = 261.848 ms) consistent with biological synaptic relaxation times.

The effect of the number of pulses on the LTP/D characteristics was then continued to be investigated. In all the LTP/D characterization tests in the electrical stimulation mode, the pulse amplitude was ±60 V, the source‐drain voltage was 0.1 V, the gate pulse width was fixed at 10 ms, and the pulse frequency was fixed at 4 Hz. From the LTP/D curves of the five cycles in Figure S10 (Supporting Information) for the half cycles of 16, 32, and 64, in which the curves were basically kept the same in shape and value, it can be seen that the synaptic device has good repeatability and stability. Figure 3g is obtained by arbitrarily selecting the two pulses in Figure S10 (Supporting Information), respectively, from which it can be clearly seen that the PSC peak increases with the number of pulses. Their single‐cycle normalized LTP/D curves were extracted as shown in Figure 3h. The date of |NL|, symmetricity, G_max_/G_min_, and *NS*_eff_ were extracted from the LTP/D curve in Figure 3h to obtain Figure 3i. |NL| is obtained from the following Equation:^[^ ^46^ ^]^

(1) $$G_{LTP}=G_{c}\;\left(1-e^{\left(-\frac{P}{L}\right)}\right)+G_{min}$$

(2) $$G_{\mathrm{LTD}}=-G_{c}\left(1-e^{\left(\frac{P_{\max}-P}{L}\right)}\right)+G_{\max}$$

(3) $$G_{c}=\left(G_{\max}-G_{\min}\right)\;/\left(1-e^{\left(-\frac{P_{\max}}{L}\right)}\right)$$

where G_LTP_ and G_LTD_ represent the conductance of the LTP/D curves, respectively; G_c_ is the fitting constant used to normalize the conductance range; L is the value of NL; P is the number of pulses applied, with P_max_ being the maximum value; and G_min_ and G_max_ denote the minimum and maximum conductance values, respectively. Symmetricity is the reciprocal of the symmetricity error and can be derived as follows:

(4) $$\mathrm{Symmetric}\;\mathrm{error}=\sum_{k=0}^{k=n}\frac{{\left(G_{N}\left(k\right)-G_{N}\left(2n-k\right)\right)}^{2}}{n}\;$$

where G_N_ (k) is the normalized conductance and n is the number of pulses in a half‐cycle. *NS*_eff_ is the number of conducting states where the ratio of ΔG to (G_max_ – G_min_)is greater than 0.5%.^[^ ^47^, ^48^ ^]^ From Figure 3i, it could be seen that the dynamic range (G_max_ / G_min_) increases gradually with the number of pulses, from 5.6 to 8.0; *NS*_eff_ decreases from 100% to 64%; LTP was smallest for 32 half‐periodic pulses, but LTD was largest. The symmetricity varies from 3 to 3.6, indicating that the symmetricity of the device is not significantly affected by the different semi‐circular pulses. These results suggest that there is a trade‐off between these performance metrics.

### 2.4 Photoelectrically Modulated Synaptic Properties

**Figure 4:**
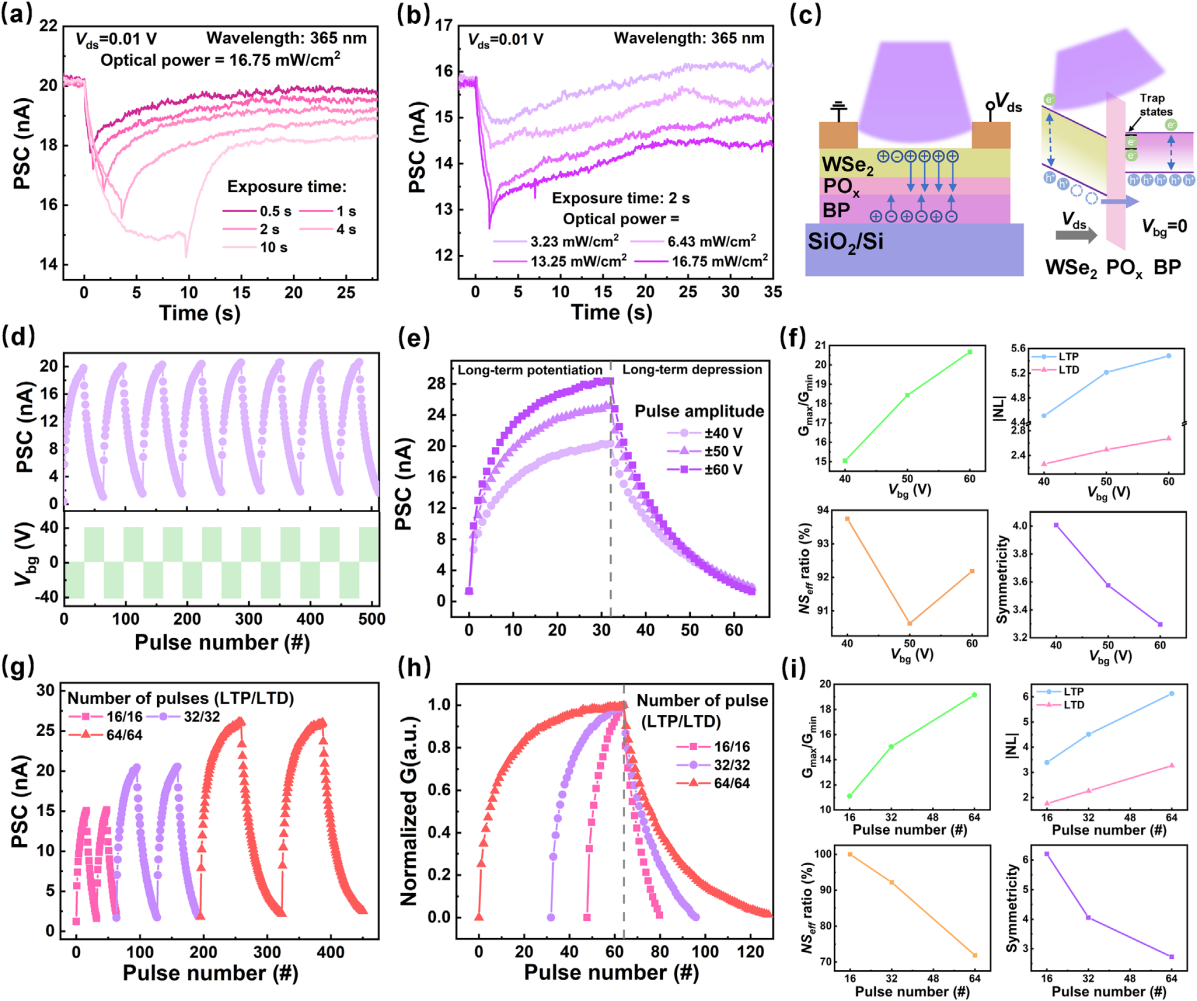
a) PSCs excited by 365 nm light pulses of different times. b) PSCs excited by 365 nm light pulses of different powers. c) Schematic diagram of the device's operation under light. d) 8‐cycle LTP/D curves (±40 V) under positive‐negative alternating *V*_bg_ pulses under light illumination. e) LTP/D curves at different electrical pulse widths (±40 V, ±50 V, ±60 V) under light illumination. f) G_max_/G_min_, |NL|, *NS*_eff_, and symmetricity values for different pulse widths under light illumination. g) Two‐cycle LTP/D characteristic curves for different pulse numbers under light illumination. h) Normalized conductance of LTP/D at different *V*_bg_ pulse numbers under light illumination. i) G_max_/G_min_, |NL|, *NS*_eff_, and symmetricity values for different pulse numbers under light illumination.

Based on the good electrical synaptic behaviors of the device, the photoelectrical synaptic behaviors of the device were further explored. In order to reduce the power consumption, the tests were performed under a 365 nm laser using the conditions of *V*_bg_ = 0 and *V*_ds_ = 0.01 V. As shown in **Figure** **4**a,b, by applying light pulses with different optical powers and exposure times, the range of PSC variation of the device increases during light irradiation and remains for a period of time after the loss of light, exhibiting persistent photocurrent (PPC) behavior.^[^ ^49^, ^50^, ^51^ ^]^ Similar to electrically modulated synapses, this process can be modeled as a transition from STP to LTP. According to Figure 4a, through the equation:^[^ ^52^, ^53^, ^54^ ^]^ E = *V*_ds_ × *I*_PSC_ × t, it can be calculated that the device could achieve a minimum power consumption of 10 pJ under 365 nm light, which is superior to most of the currently reported artificial synaptic devices, as shown in Figure S11 (Supporting Information) for the comparison of their power consumption.^[^ ^55^, ^56^, ^57^, ^58^, ^59^, ^60^, ^61^, ^62^ ^]^ Stimulating the device with two consecutive light pulses as shown in Figure S12 (Supporting Information), where the pulse width is 1 s, the pulse interval time is 1 s, and the pulse power is 16.75 mW cm^−2^, the PSC induced by applying the second light pulse is larger than that induced by the first light pulse, which is similar to the PPF behavior observed in biological synapses. The PSC was also modulated by varying the number of light pulses, as shown in Figure S13 (Supporting Information), with the increase of the number of light pulses, the PSC had a tendency to transition from STP to LTP, and the device was gradually saturated with the photogenerated carriers that could be stored, so that there was little change in the last two pulses.

As shown in Figure 4c, the device operates under 365 nm illumination as follows: the BP generates a large number of photogenerated carriers due to an absorption peak in the violet band (e.g., 365 nm), while the WSe_2_ generates only a small number of carriers due to the PPC. Since WSe_2_ is a p‐type semiconductor, its channel layer has holes as majority carriers, which accumulate in the valence band and form transmission channels under forward bias. At the same time, photogenerated electrons generated in the BP are attracted to the BP/PO_x_ interface, but due to the presence of localized defect traps in the PO_x_ layer as well as a high hole‐injection barrier, the electrons are trapped at the interface, which triggers a photogenerated gate‐pressure effect through the recirculation of holes in the channel. This effect leads to a dramatic decrease in the thickness of the hole‐injection barrier of the PO_x_ layer, allowing hole tunneling enhancement to be achieved with a small forward bias. Thus, the PO_x_ layer acts as an “photoelectronic valve” for hole injection through photonic modulation, in which the incident photon modulates the conductive state of the device by triggering the carrier dynamics described above.^[^ ^29^ ^]^

LTP/D characteristics are key synaptic properties that have a significant impact on the overall performance of the neural system. In hardware neural networks, it is crucial to be able to simulate and implement LTP/D characteristics, as this determines the learning ability and adaptability of hardware neural networks.^[^ ^63^, ^64^, ^65^ ^]^ Based on the device's excellent photo‐synaptic plasticity and output characteristics with adjustable *V*_bg_ under 365 nm illumination (Figure S14, Supporting Information), we investigated the effect of electrical pulse width under illumination on the LTP/D characteristics. In all subsequent LTP/D characterization tests, the gate pulse width was fixed at 100 ms, the pulse frequency was fixed at 4 Hz, and the source‐drain voltage was 0.01 V. Figure 4d and Figure S15 (Supporting Information) show the LTP/D curves for multiple cycles under light‐illuminated positive‐negative alternating *V*_bg_ pulses (±40 V, ±50 V, and ±60 V), and the shapes and values of the curves basically remain unchanged, indicating that the device has good repeatability. The effect of different electrical pulse widths (±40 V, ±50 V, ±60 V) on the LTP/D characteristics is tested in Figure 4e and evaluated in Figure 4f for a fixed 32‐pulse condition under illumination. The G_max_/G_min_ increased from 15.0 to 20.7 with increasing pulse amplitude; the |NL| increased approximately linearly; the *NS*_eff_ decreased but was all above 90%; and the symmetricity decreased approximately linearly.

The effect of the number of electrical pulses on the LTP/D characteristics of the device under light illumination was continued. Based on the effect of pulse width on the LTP/D characteristics, the two‐cycle LTP/D characterization was selected to be performed with different numbers of pulses (16, 32, and 64) at a pulse amplitude of 40 V under comprehensive consideration. From the LTP/D curves of multiple cycles at 16, 32, and 64 half‐cycles in Figure S16 (Supporting Information), it can be seen that the shapes and values of the curves remain basically the same, indicating that the device has good repeatability. Figure 4g is obtained by arbitrarily selecting two pulses in Figure S16 (Supporting Information), respectively, from which it is clear that the PSC peak increases with the number of pulses. To further evaluate the effect of the number of pulses on the LTP/D performance, the LTP/D curves at a single cycle were extracted, and their PSCs were converted to conductance and normalized as shown in Figure 4h. The values of |NL|, symmetricity, G_max_/G_min_, and *NS*_eff_ are extracted from the LTP/D curves of Figure 4h, and they are calculated as shown in Figure 4i. As the number of pulses increases, the G_max_/G_min_ increases from 11.1 to 19.2; the |NL| increases approximately linearly; the *NS*_eff_ decreases from 100% to 71.9%; and the symmetricity decreases approximately linearly from 6.2 to 2.7.

### 2.5 Simulation of Visual Memory Behavior

**Figure 5:**
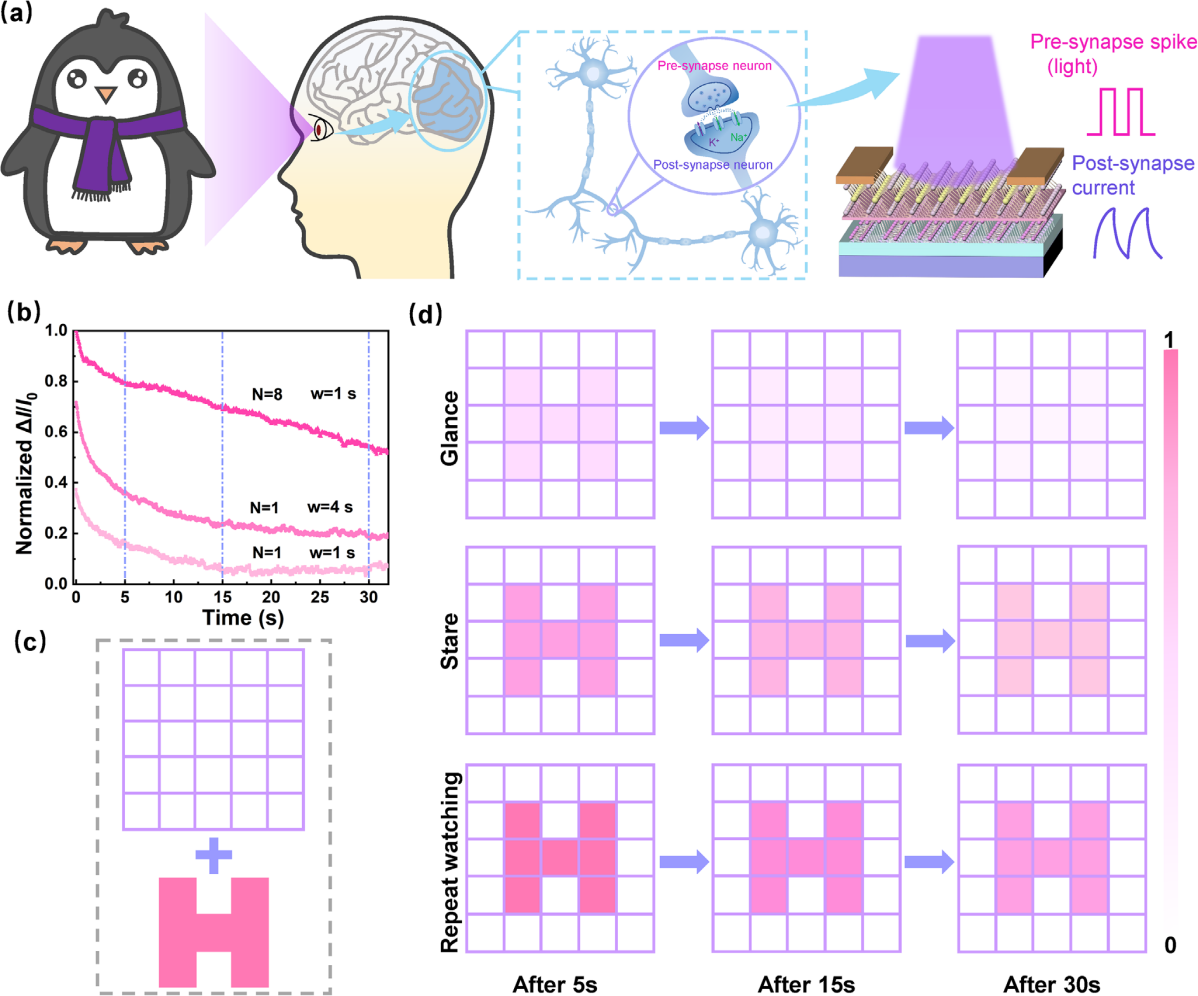
a) Schematic diagram of the device simulating the visual memory behavior of the human eye. b) Normalized Δ*I*/*I*_0_ values after the action of pulsed laser with different pulse widths and number of pulses (λ = 365 nm, P_in_ = 16.75 mW cm^−2^, *V*_ds_ = 0.01 V). c) Background and input signal “H”. d) Visual persistence after illumination (365 nm, 16.75 mW cm^−2^) for 5, 15, and 30 s with different pulse widths/numbers.

Human visual perception is fundamentally dependent on the transient retention and neurological processing of photonic stimuli, a biological process initiated through retinal phototransduction where external light signals are converted into spatiotemporal neural patterns for cortical integration.^[^ ^66^, ^67^ ^]^ This cascade mechanism underlies the phenomenon of visual persistence, a key sensory memory mechanism in which sensory afterimages are retained in the visual cortex after the stimulus is removed, enabling temporal continuity in perceptual experience.^[^ ^68^ ^]^ Similar to human visual persistence, the retention ability triggered by optical stimuli exhibits a slow decay characteristic when the stimulus is removed, a phenomenon that can be used to model visual persistence in the nervous system.^[^ ^69^ ^]^ **Figure** **5**a then depicts the corresponding diagram of the device modeling this process. Based on the above photoelectric synaptic properties, human visual memory behavior was simulated by increasing illumination time, number of light pulses, and time after illumination (Figure 5b; Figure S17, Supporting Information). The results showed that the normalized *ΔI/I_0_* increased with increasing pulse width and number of pulses, and the decay slowed down after illumination. In Figure 5c, the character “H” was used as an input signal, and an image of the character was formed immediately after stimulation. In Figure 5d, corresponding to Figure 5b and Figure S17 (Supporting Information), a short duration of illumination, a long duration of illumination, and repeated illumination are used to simulate glancing, staring, and repeated watching, respectively. In Figure 5d, when the device receives the briefly presented letter “H”, a recognizable current change occurs, similar to a quick glance. Even 30 s after the end of the visual signal, the “H” shape remains, confirming the visual memory effect. The normalized *ΔI/I_0_* decreased gradually with time, which was consistent with the experimental results. Visual memory was significantly improved after a prolonged stare compared with a brief glance. Even in the images after 5 and 30 s after the end of the visual signal it could be seen that retention was significantly enhanced. And repeated viewing has an even more pronounced effect, which suggested that memory could be effectively preserved by repeated viewing. Thus, the proposed floating‐gate photoelectron synaptic device has the function of perceiving and memorizing images in response to light stimuli.

### 2.6 Neuromorphic Computational Simulation

**Figure 6:**
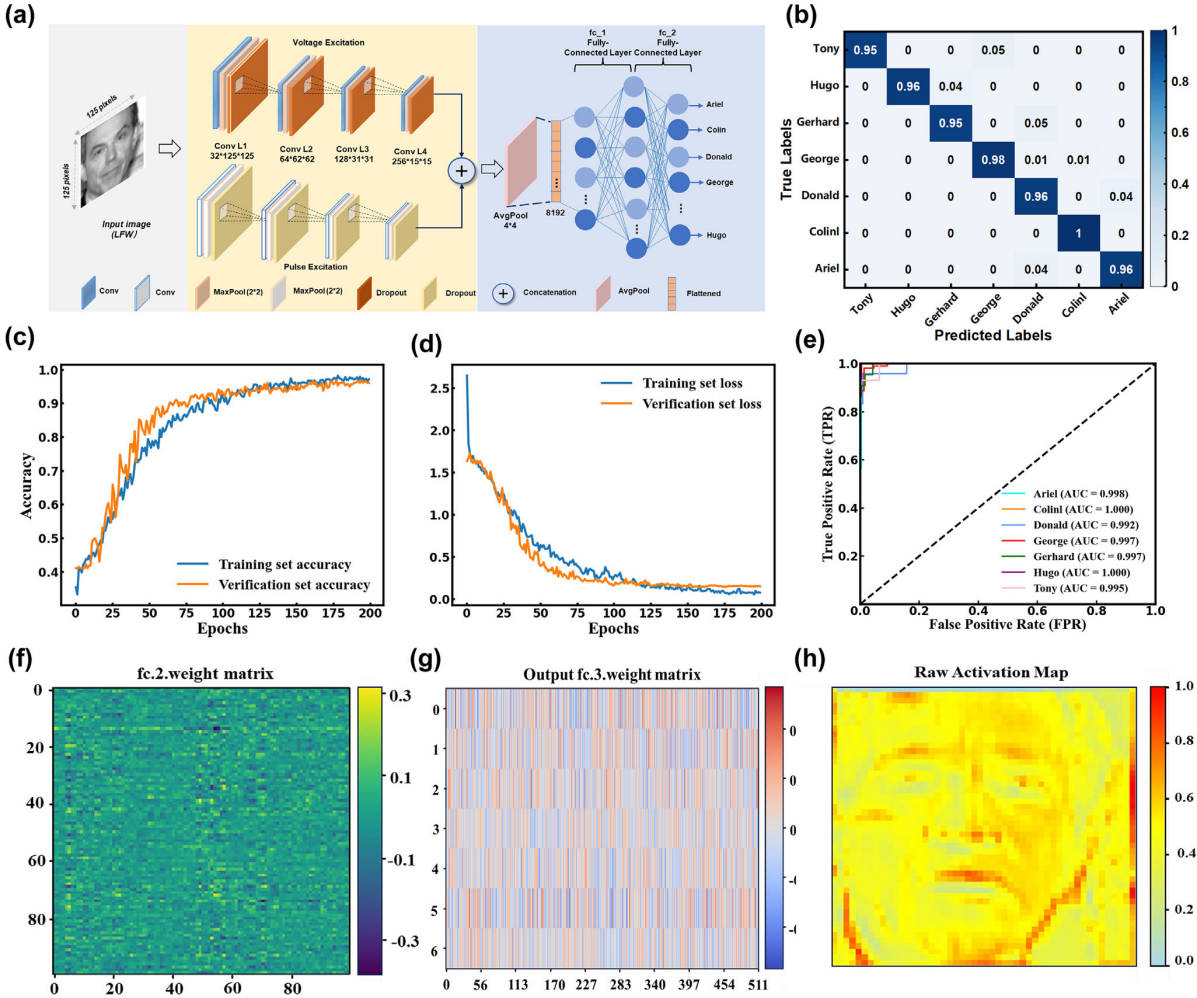
a) Extended CNN model with two input channels, voltage‐excited and pulse‐excited, each with four convolutional layers and a 3‐layer fully connected neural network. b) Confusion matrix of the seven classification results. c) Accuracy of CNN model training and test sets. d) Loss curve of the CNN model. e) ROC curves for different samples. f) Weight matrices of the first 100 input neurons and output neurons of the second fully connected layer. g) Weight matrices of different output categories of the fully connected layer. h) Activation feature map of the output after fusion of convolutional layers.

In order to evaluate the visual processing capability and recognition accuracy of this emerging artificial synaptic device, a modified CNN model is fed by two channels, voltage‐excited and pulse‐excited, and different convolutional layers are designed using the parameters of the device, and then fused (as shown in **Figure** **6**a). The constraints (LTP/D curves) simulate the regulation mechanism of synaptic strength in biological neurons, which is used to regulate the nonlinear properties of the activation function; *NS*_eff_ determines the sensitivity of the neuron, with higher values indicating that the activation requires stronger inputs, which directly affects the feature selectivity in the network; G_max_/G_min_ affects the standard deviation of the initialization of the weights to control the range of distribution of the initial weights, and this dual‐path design simulates the different response patterns of different types of neurons to stimuli in a biological nervous system. Face recognition on the LFW dataset is applied with the architecture of this CNN, which consists of 7 classes of grayscale images of faces, where images of size 125 × 125 pixels are fed into the network. The voltage path utilizes the voltage parameters to design four dynamic convolutional layers; the impulse path utilizes the impulse parameters to design also four dynamic convolutional layers; the outputs of the subsampling layers are passed to a series of fully connected layers for feature fusion using a two‐layer structure that allows the network to learn nonlinear combinations of features. The final layer outputs a vector of size 7, corresponding to a probability distribution over a 7‐bit category. This hierarchical CNN structure involves repeated convolution and subsampling steps for different stimulus modalities, simulating the response properties of different types of neurons, and complementary feature extraction capabilities allow the model to extract the complex features required for accurate face recognition. The confusion matrix illustrates the performance of the 10‐class classification model, where the rows represent the actual classes and the columns represent the predicted classes (Figure 6b). The diagonal values indicate the class‐specific accuracies, and the classification accuracies for the different classes exceed 95%, reflecting the robust model performance of the neural device. Figure 6c,d for the training and test sets of the model show the change process of accuracy and loss in the training and test sets during the training process of the model, and the whole training process shows a high degree of stability, and the model tends to stabilize at 200 epochs, with a classification accuracy of 96.90%. The values of the area under the ROC curve, AUC, are also very high, both of which reach 99% (Figure 6e), indicating the superior classification performance of the model. The weight distribution map of the fully connected layer (Figure 6f) is responsible for integrating the features extracted from the voltage path and the impulse path for further transformation. Each row represents an output neuron and each column represents an input feature, showing the extent of its influence on all output neurons. The structured stripe pattern indicates that the network learns the correlation between the features. Whereas the fully‐connected output layer weight matrix shows a more even color distribution, represented by different colors from high (red) to low (color) (Figure 6g), this shift reflects the optimization of the weights, and the narrower range of weights indicates that the CNN has adapted and improved the connections to capture the underlying features that improve performance. The reduction in post‐training variability suggests that the model has selectively strengthened the relevant connections and reduced the irrelevant weights, thereby improving its efficiency and classification accuracy. In order to validate the convolutional layer in extracting key features, the fused activation feature maps (Figure 6h) reveal the image regions that the neural network “focuses” on when making classification decisions. It highlighting the network's ability to focus on semantically critical facial regions (such as the eyes, nose, and mouth), thereby mimicking human visual perception. This attention distribution is closely associated with the high AUC values. The model achieves an accuracy of 96.9% on the LFW dataset, which involves unconstrained variations in lighting, pose, and expression. This significantly exceeds the complexity of simpler datasets such as MNIST and CIFAR‐10, where Li et al.^[^ ^70^ ^]^ reported over 95% accuracy for colored facial image recognition, and Chen et al.^[^ ^71^ ^]^ reported 97.1% on MNIST but only 85.5% on CIFAR‐10. The heat map is more focused on the key areas of facial recognition (features such as eyes, mouth, contours, etc.). These results show that the model effectively improves the recognition accuracy and feature learning ability by incorporating voltage and pulse excitation feature extraction mechanisms. It performs well in optimizing weight distribution, enhancing critical feature extraction, and stable training. In the Supporting Information, Tables S1 and S2 (Supporting Information) respectively present the basic performance and specific algorithm usage comparisons of this device with other devices. This result not only highlights the excellent basic performance of this floating‐gate device and its advantages as a dual‐path artificial synaptic device.

## 3 Conclusion

In summary, we have developed an innovative BP/PO_x_/WSe_2_ floating‐gate transistor architecture by strategically integrating spontaneously oxidized BP derivatives, where BP is utilized as a charge‐trapping layer and its intrinsic oxide (PO_x_) as a dielectric component. This heterostructure design capitalizes on the synergistic material properties and interfacial engineering to achieve breakthrough nonvolatile memory characteristics, including an exceptional current on/off ratio of ≈10⁵ and an expansive 73 V memory window. Performance exploration of the device system demonstrated its multimodal synapse simulation capability, successfully simulating a rich set of biological synaptic properties, including STP, LTP, and bi‐directional weight updating (LTP/D) in both electrical and photoelectronic modulation modes. Particularly noteworthy is the photoelectronic modulation mode, which not only enables artificial visual memory simulation but also achieves ultra‐low energy consumption of 10 pJ for the smallest synaptic event, a performance metric superior to most reported photoelectronic neuromorphic devices. Taking advantage of these dual‐mode benefits, we further designed a bionic dual‐path convolutional neural network to synergistically process optical pulse sequences and electrically gated signals, achieving a recognition accuracy of 96.9% on the challenging LFW face dataset. This device‐system co‐optimization framework establishes a new material‐design paradigm for vision‐processing neuromorphic electronics, offering tangible hardware solutions for next‐generation artificial intelligence systems requiring adaptive sensory‐computational integration, particularly in bio‐vision applications demanding high parallelism and energy efficiency.

## 4 Experimental Section

### Materials

The BP and WSe_2_ used in this experiment were purchased from Shanghai Onway Technology Co., Ltd.

### Device Fabrication

The floating‐gate structures were made by sequentially stacking BP and WSe_2_ obtained by mechanical stripping using a PDMS (polydimethylsiloxane)‐assisted dry transfer process on a 300 nm SiO_2_/Si substrate. First, the BP was transferred to the cleaned silicon substrate by the dry transfer method, and the WSe_2_ was stacked on it by the dry transfer method after standing in air for 20 min. Next, PMMA A4 (polymethylmethacrylate) was spin‐coated onto the fabricated floating‐gate structure and patterned using electron beam lithography. Finally, Ti/Au (10/50 nm) electrodes were deposited using magnetron sputtering.

### Measurement and Characterization

The atomic structural features of the BP/PO_x_/WSe_2_ floating‐gate structure were examined using a high‐resolution transmission electron microscope (Talos F200X, FEI), and the composition and elemental distribution of the heterojunction were analyzed by EDS mapping on a high‐resolution transmission electron microscope. Morphological features of the floating‐gate structures were investigated by optical microscopy (BX51, OLYMPUS). The thickness of the synaptic devices was determined by atomic force microscopy (Dimension ICON, BRUKER). X‐ray photoelectron spectroscopy (XPS) tests were performed using a PHI 5000 Versaprobe II instrument with an aluminum Kα radiation source at a wavelength of 1486.6 eV. Raman spectroscopy measurements were performed using a Raman spectrometer system (LABRAM HR, HORIBA Jobin Yvon) at a 532 nm laser source. The electrical transport properties and synaptic characteristics of the device were tested at room temperature under vacuum using a Keithley 2612B instrument. For light pulse measurements, the LAMPLIC UVEC‐4II point light source with multiple irradiation heads was used.

## Conflict of Interest

The authors declare no conflict of interest.

## Author Contributions

Y.X.Z. and W.X.L. contributed equally to this work. Z.M.Z. and L.B.G. initiated the project. Y.X.Z. Conducted photoelectric synapses testing. Z.P.Y., Z.S.H., R.B.Z. T.L.Z. Y.W.L., Y.L., and B.F. analyzed the experimental data. Z.H.H. and W.H.H. performed and supervised the algorithm deployment. Y.X.Z., W.X.L., Z.H.H., and W.H.H. wrote the manuscript. All the authors joined in the discussion and analysis of experimental data and revision of the manuscript.

## Supporting information



Supporting Information

## Data Availability

The data that support the findings of this study are available from the corresponding author upon reasonable request.
