# Supplementary material for: A Floating‐Gate Photoelectric Synaptic Transistor Utilizing BP/POx/WSe2 Heterostructure for Neuromorphic Visual Processing
Source: Adv Sci (Weinh). 2025 Sep 14;12(45):e10063. doi: 10.1002/advs.202510063 (PMC12677613; doi:10.1002/advs.202510063)
Supplement: Supplementary file 1 — Supporting Information [file ADVS-12-e10063-s001.docx]

**A Floating-Gate Photoelectric Synaptic Transistor Utilizing BP/POx/WSe₂ Heterostructure For Neuromorphic Visual Processsing**

Yuxuan Zeng^1,2#^, Wenxing Lv^3#^, Zehai Hou^1^, Weihua Huang^1^, Zhipeng Yu^2^, Zishuo Han^2^, Rongbin Zhan^2^, Tianle Zeng^2^, Yawen Luo^2^, Yu Lin^2^, Weiming Lv^2^, Bin Fang^2^, Zhongming Zeng^2,*^, Lianbo Guo^1,*^

**Supporting Information**


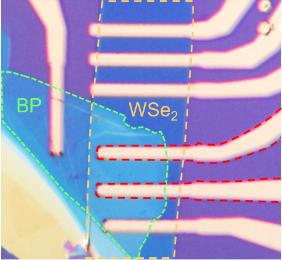


**Figure S1**. The optical image of the floating-gate device.


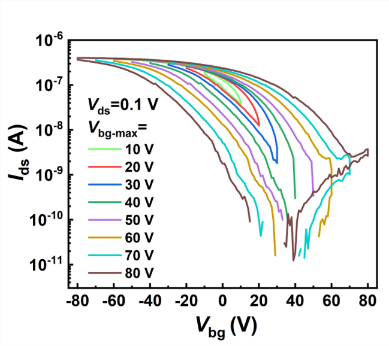


**Figure S2**. The logarithmic form graph of the transfer curve of this floating-gate device.


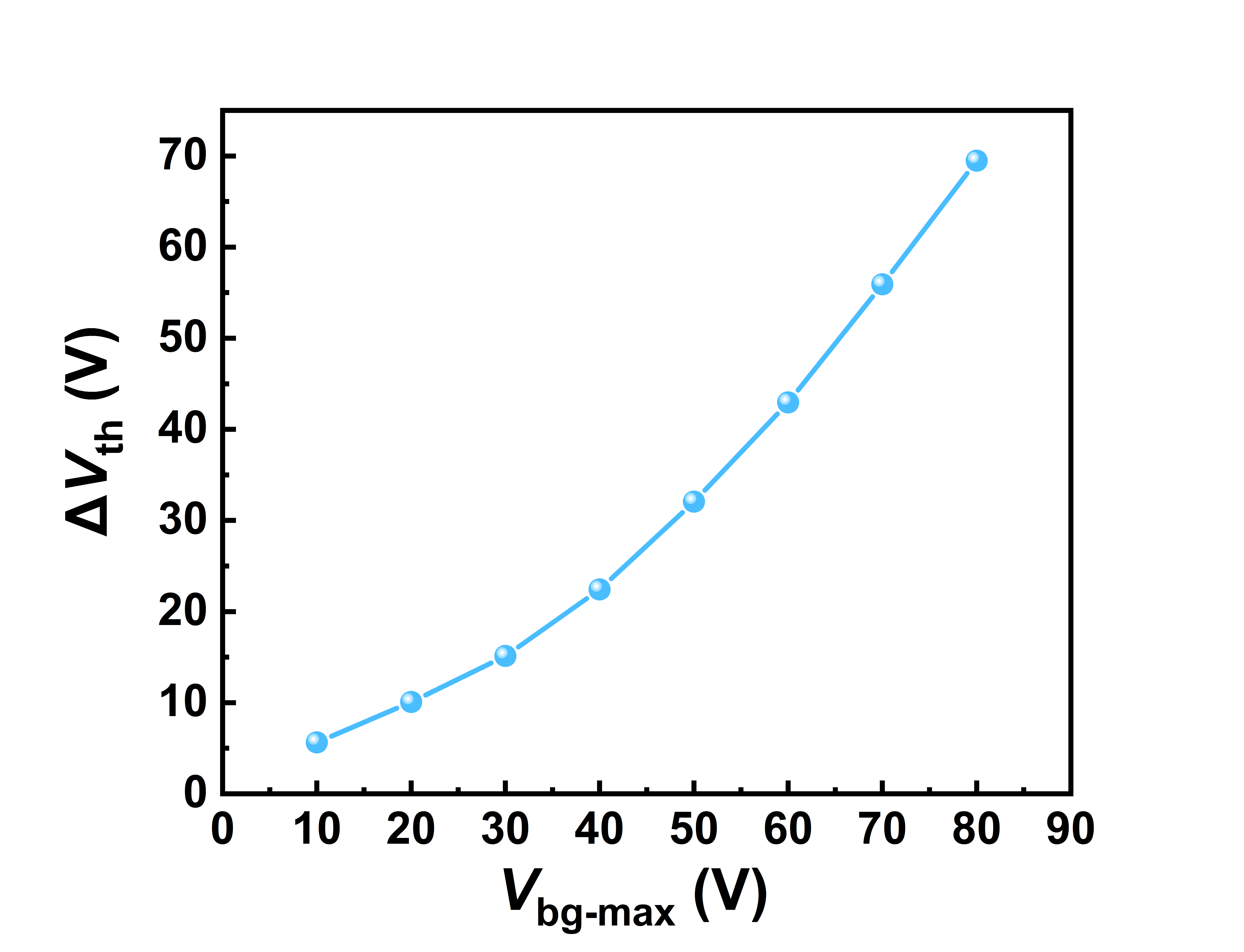


**Figure S3**. Transfer characteristic curve when the maximum value of grid voltage scanning range *V*_bg-max_ was 10 ~ 80 V.


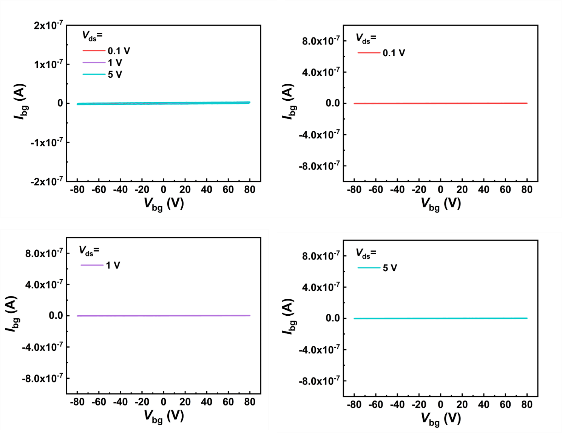


**Figure S4**. The *I*_ds_-*V*_bg_ curve graph of this floating-gate device.


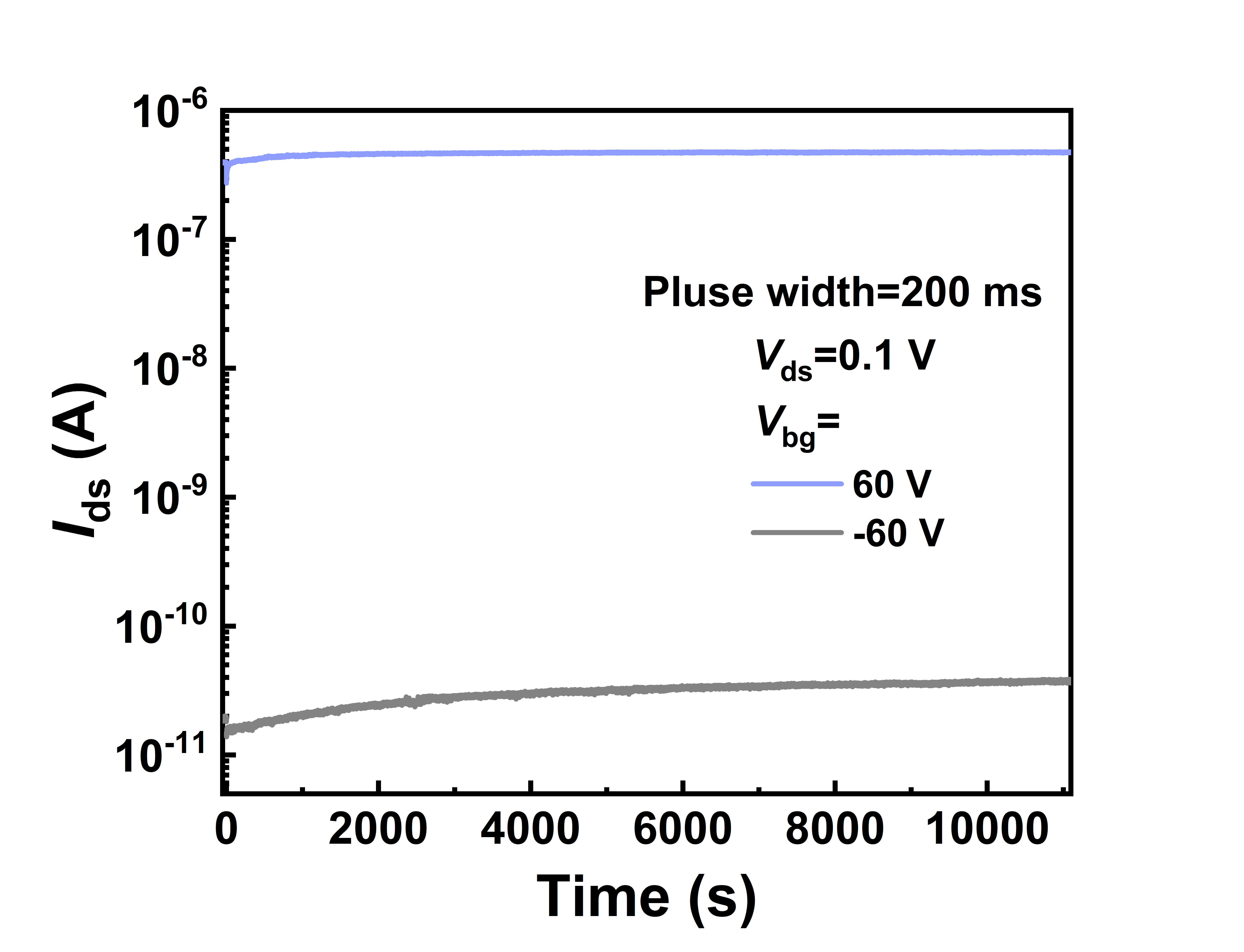


**Figure S5**. The retained characteristics of this floating-gate device.


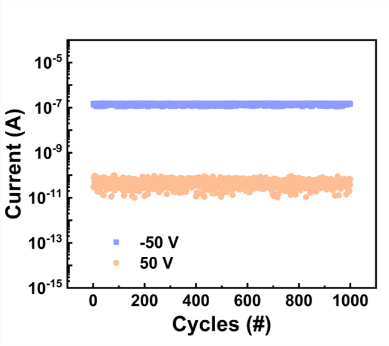


**Figure S6**. The endurance characteristics of this floating-gate device.


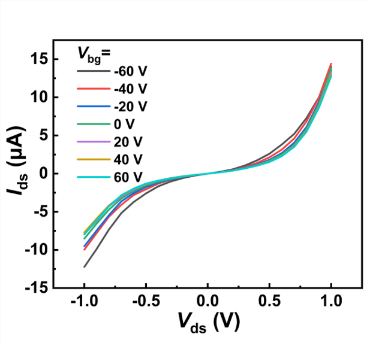


**Figure S7**. Output characteristic curve of WSe_2_ field-effect transistor.


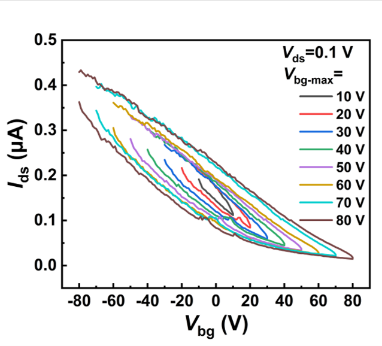


**Figure S8**. Transfer characteristic curve of WSe_2_ field-effect transistor.


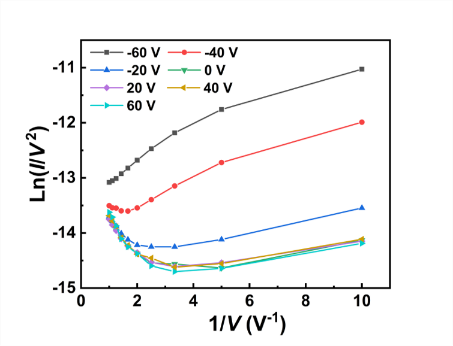


**Figure S9**. The value (ln(*I*_ds_*/**V*_ds_^2^)) as a function of *V*_ds_^-1^ in the positive *V*_ds_ region.


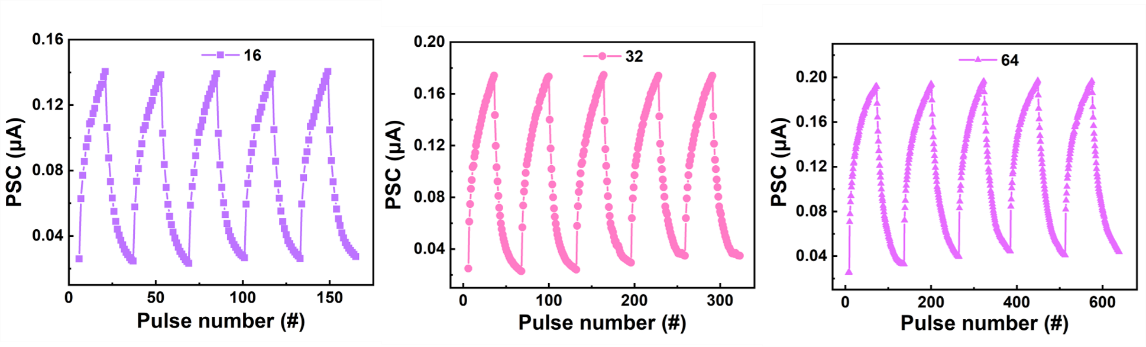


**Figure S10**. LTP/D curves for five periods at 16, 32, and 64, respectively.


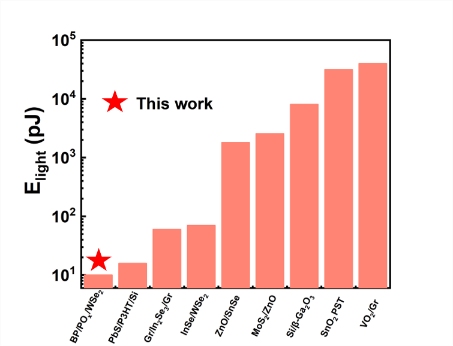


**Figure S11**. Comparison of power consumption of different photoelectric synaptic devices was plotted.


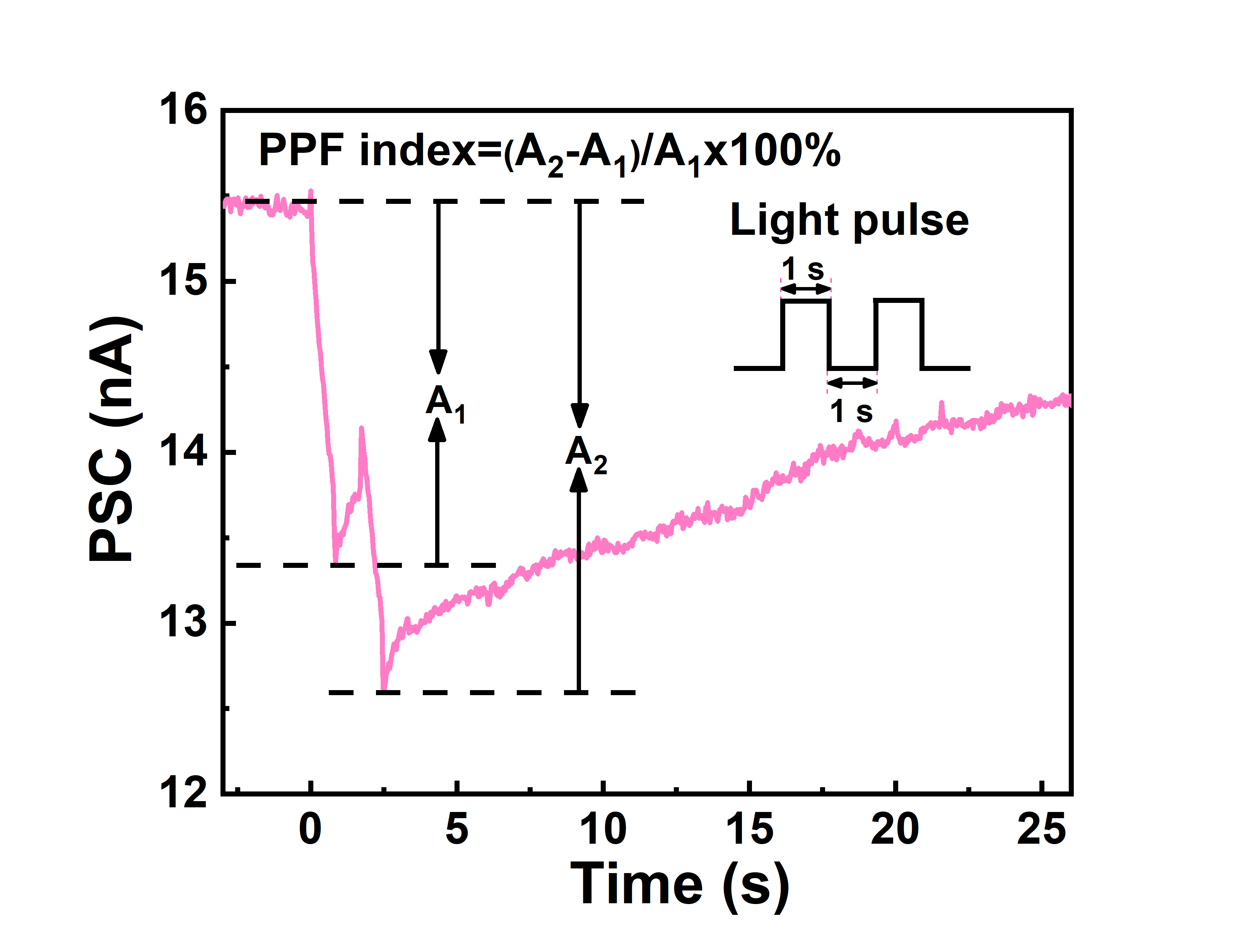


**Figure S12**. PPF under light pulses.


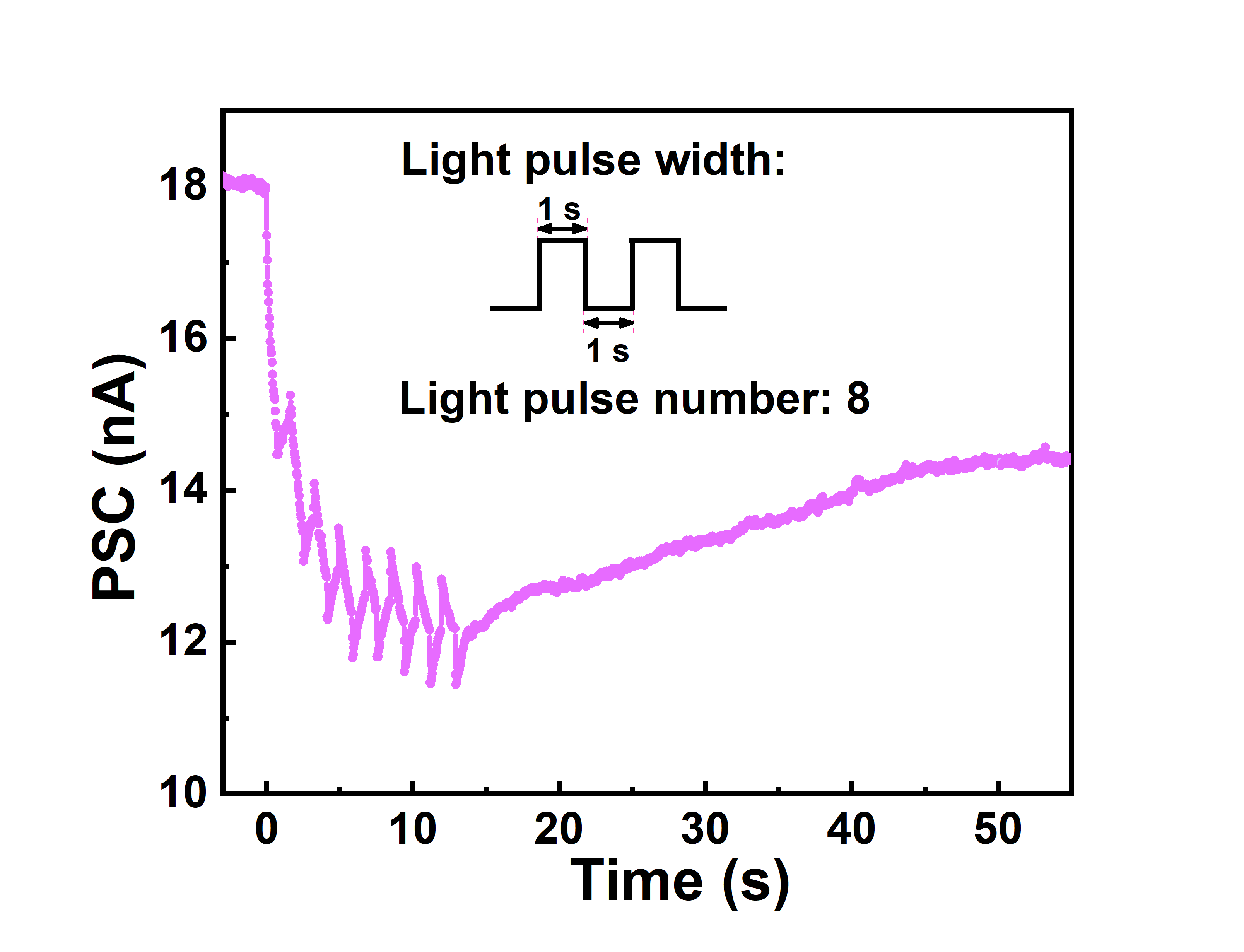


**Figure S13**. PSC by 8 number of light pulses.


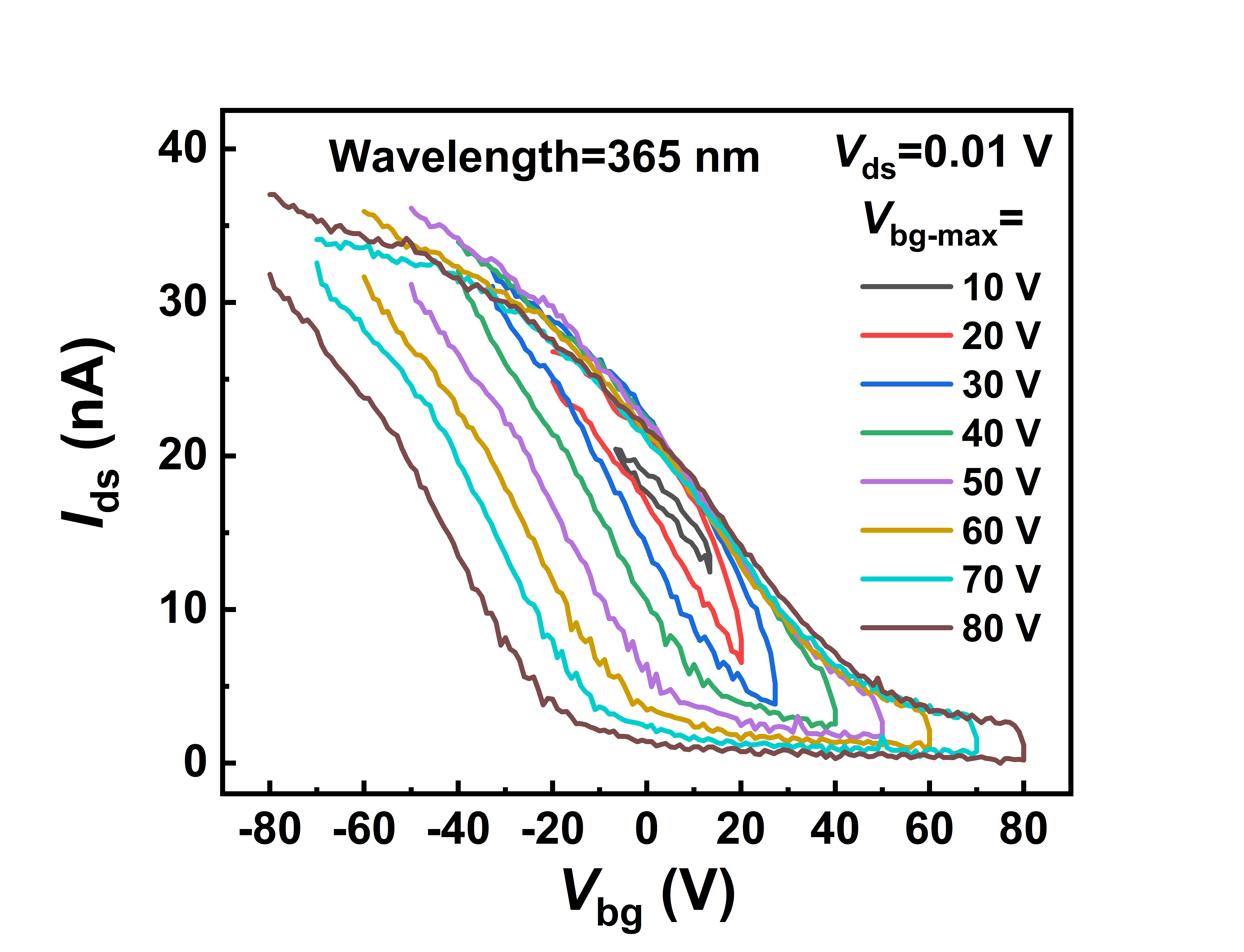


**Figure S14**. Transfer characteristic curve controlled by *V*_bg_ under 365 nm illumination.


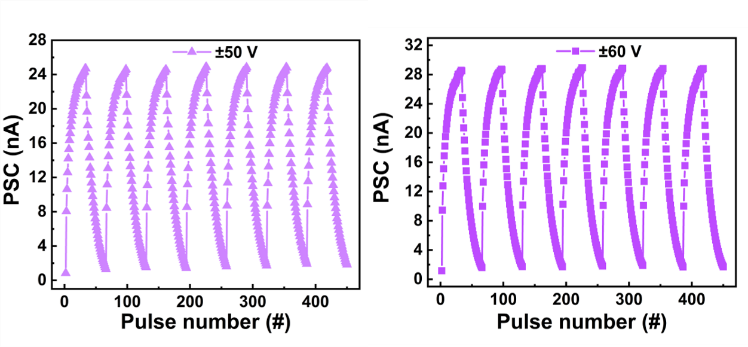


**Figure S15**. 7-cycle LTP/LTD curves (±50 V and ±60 V) under positive-negative alternating *V*_bg_ pulses under light illumination.


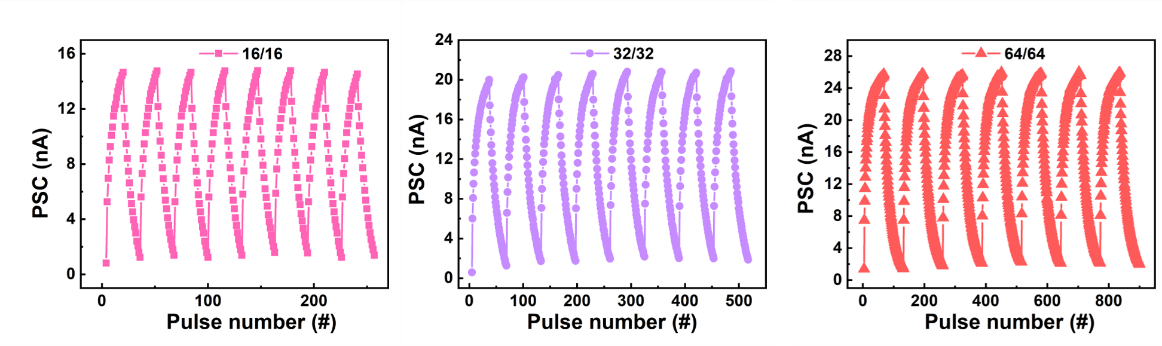


**Figure S16**. Multi-cycle LTP/LTD characteristic curves for different number of pulses under light illumination.


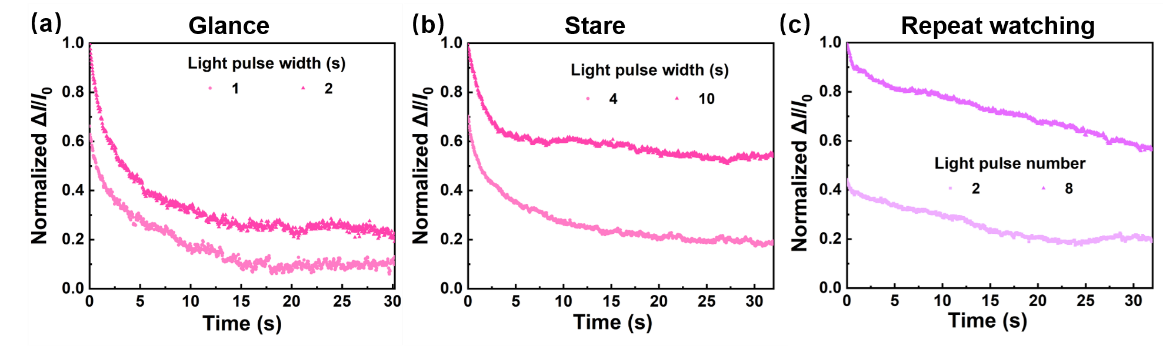


**Figure S17**. Normalized *∆I/I_0_* after a single laser pulse with the pulse width (a) below and (b) above 2 s, and (c) pulse numbers from 2 of 8 (pulse width: 1 s).

**Table S1. Comparison of key performance characteristics of different 2D floating-gate devices**

| Device structure | On/off ratio | retention rate | Memory  ratio | Cycle  endurance | Minimum power consumption | Function | Image recognition accuracy | Ref |
| --- | --- | --- | --- | --- | --- | --- | --- | --- |
| MoS_2_/h-BN/Gr | 10^3^ | 10000 | 43.8% | / | / | Memory/ Logical operation | / | 1^[1]^ |
| MoS_2_/h-BN/Au | 10^5^ | 1000 | 33.3% | / | / | Memory/ Logical operation | / | 2^[2]^ |
| MoTe_2_/h-BN/Gr | 10^3^ | 1800 | 54.2% | / | / | Memory | / | 3^[3]^ |
| WSe_2_/h-BN/CIPS/Gr | 10^5^ | / | 50.0% | 100 | 20 pJ | Artificial Synapse | 84.9% | 4^[4]^ |
| WSe_2_/h-BN/CIPS | 10^6^ | 10000 | 41.7% | / | / | Logical operation | / | 5^[5]^ |
| MoS_2_/β-Ga_2_O_3_/Gr | 10^6^ | 1000 | 78.44% | 1000 | 254.4 pJ | Memory/ Artificial Synapse | 91.07% | 6^[6]^ |
| WSe_2_/PO_x_/BP | 10^5^ | 11000 | 45.6% | 1000 | 10 pJ | Artificial Synapse | 96.9% | This work |

**Table 2. Performance Benchmarking**

| Reference (Year) | Device Structure | Input Modality | Task/Dataset | Accuracy (%) | Key Advantage | Ref |
| --- | --- | --- | --- | --- | --- | --- |
| Chueh et al. (2023) | WSeOx/WSe_2_ memristor | Electrical | MNIST digits | 87 | Ultra-low voltage (4 mV) | 7^[7]^ |
| Chen et al. (2023) | All-analog optoelectronic ACCEL | Optoelectronic | MNIST/CIFAR | 97.1/85.5 | 74.8 Peta-OPS/W | 8^[8]^ |
| Li et al. (2024) | Plasmon-enhanced WSe_2_ | Optoelectronic | Color image recognition | >95 | Ultra-low energy | 9^[9]^ |
| **Our work (2025)** | **BP/PO_x_/WSe_2_ dual-path CNN** | **Optoelectronic** | **LFW face recognition** | **96.9** | **High accuracy in complex tasks** | **—** |

**References**

[1] H. Wu, Y. Cui, J. Xu, Z. Yan, Z. Xie, Y. Hu, S. Zhu, *Nano Lett.* **2022**, *22*, 2328.

[2] D. Zeng, R. Ding, G. Liu, H. Lu, M. Zhang, Z. Xue, Z. Tian, Z. Di, *Adv. Electron. Mater.* **2024**, *10*, 2300621.

[3] S. Wang, G. Geng, Y. Sun, S. Wu, X. Hu, E. Wu, J. Liu, *Nano Res.* **2022**, *15*, 6507.

[4] J. Bai, D. He, B. Dang, K. Liu, Z. Yang, J. Wang, X. Zhang, Y. Wang, Y. Tao, Y. Yang, *Adv. Mater.* **2024**, *36*, 2401060.

[5] A. Ram, K. Maity, C. Marchand, A. Mahmoudi, A. R. Kshirsagar, M. Soliman, T. Taniguchi, K. Watanabe, B. Doudin, A. Ouerghi, S. Reichardt, I. O’Connor, J.-F. Dayen, *ACS Nano* **2023**, *17*, 21865.

[6] J. Zeng, L. Hu, Y. Pan, Z. Huang, H. Sun, J. Zeng, X. Xie, H. Chen, *Adv. Funct. Mater.* *n/a*, 2508292.

[7] M. Chaudhary, T.-Y. Yang, C.-T. Chen, P.-C. Lai, Y.-C. Hsu, Y.-R. Peng, A. Kumar, C.-H. Lee, Y.-L. Chueh, *Adv. Funct. Mater.* **2023**, *33*, 2303697.

[8] Y. Chen, M. Nazhamaiti, H. Xu, Y. Meng, T. Zhou, G. Li, J. Fan, Q. Wei, J. Wu, F. Qiao, L. Fang, Q. Dai, *Nature* **2023**, *623*, 48.

[9] T. Zhang, X. Guo, P. Wang, X. Fan, Z. Wang, Y. Tong, D. Wang, L. Tong, L. Li, *Nat. Commun.* **2024**, *15*, 2471.
